# Enzyme-catalyzed allylic oxidation reactions: A mini-review

**DOI:** 10.3389/fchem.2022.950149

**Published:** 2022-08-15

**Authors:** Maoyao Wang, Xiaojian Zhou, Zhongqiang Wang, Yongzheng Chen

**Affiliations:** ^1^ Key Laboratory of Biocatalysis and Chiral Drug Synthesis of Guizhou Province, Green Pharmaceuticals Engineering Research Center of Guizhou Province, Zunyi Medical University, Zunyi, China; ^2^ Key Laboratory of Basic Pharmacology of Ministry of Education, Joint International Research Laboratory of Ethnomedicine of Ministry of Education, Zunyi Medical University, Zunyi, China

**Keywords:** biocatalysis, allylic oxidation, C–H activation, oxyfunctionalization, UPO, P450

## Abstract

Chiral allylic oxidized products play an increasingly important role in the pharmaceutical, agrochemical, and pharmaceutical industries. Biocatalytic C–H oxyfunctionalization to synthesize allylic oxidized products has attracted great attention in recent years, with the ability to simplify synthetic approaches toward complex compounds. As a result, scientists have found some new enzymes and mutants through techniques of gene mining and enzyme-directed evolution in recent years. This review summarizes the recent developments in biocatalytic selective oxidation of olefins by different kinds of biocatalysts.

## Introduction

**FIGURE 1:**
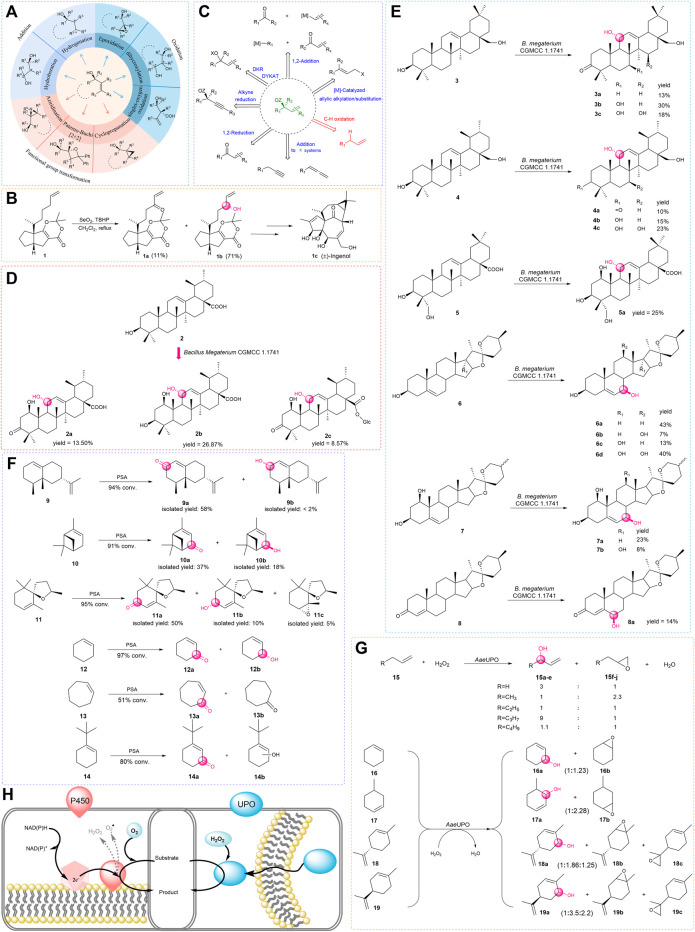
**(A)** Diastereoselective transformations of allylic alcohols. **(B)** Total synthesis of (±)-Ingenol. **(C)** Catalytic asymmetric access to enantio-enriched allylic alcohols. **(D,E)** Biotransformation of triterpenes and steroids by *Bacillus megaterium*. **(F)** Biocatalytic allylic oxidation by PSA. **(G)** Epoxidation competes with allylic hydroxylation in most cases. **(H)** P450s and UPOs are compared. (The red on the left is P450s which rely on auxiliary proteins and cofactors, the blue on the right is UPOs which require only hydrogen peroxide).

As the demand for oxygenated allylic intermediates in food, flavor and fragrance, cosmetics, and pharmaceuticals is increasing, the significance of this type of compound is gradually revealed (Casarano et al., 2005; Fanani et al., 2021; Raffo et al., 2012; Venancio et al., 2021; Ke et al., 2021). The reaction that converts alkenes to allyl alcohols has recently attracted particular interest. Due to containing two functional groups, including the hydroxyl group and olefins, this type of compound can participate in a variety of reactions such as oxidation, reduction, esterification, and addition (Sharpless and Michaelson., 1973; Church and Andersson., 2008; Pellissier, 2008) and can also be used for subsequent functional group transformation (Adam et al., 2000; Müller and Fruit, 2003; Hernández-Toribio et al., 2011) (Figure 1A). In addition, there is an increasing number of examples of allylic oxidation catalyzed by transition metals and co-oxidants to produce useful natural products; among them, the selenium and chromium reagents have been widely applied in natural product syntheses (Nakamura and Nakada, 2013). For example, (±)-Ingenol (1c) was synthesized with the use of *tert*-butyl hydroperoxide (TBHP) as a catalyst for allylic oxidation to prepare key alcohol intermediate (Figure 1B).

In the past few decades, there are several strategies available for the oxidation of allyl to allyl alcohols or allyl esters; both enzymatic and traditional organic chemical syntheses have made great contributions to the field (Lumbroso et al., 2013; Dong et al., 2018). With the development of the technology, efforts have been put together to set a goal to achieve attractive green, step- and atom-economical, and sustainable methods (Kim and Li. 2020). For this aim, enzyme-catalyzed oxidation with molecular oxygen and incorporation of the oxygen atom(s) into the allyl molecule has been emerging to be one of the most attractive research areas (Hamilton, 1974; Sono et al., 1996) (Figure 1C). First of all, this method generally avoids the use of toxic metals or expensive complex ligands and usually offers remarkable chemo-, regio-, and stereoselectivity (Jiang, 2020). Furthermore, enzymatic oxygenation is usually performed under mild reaction conditions, therefore avoiding the use of harsh conditions such as high temperature and pressure (Blank et al., 2010).

Combining the importance of allyl alcohol products with the advantages of enzyme-mediated catalysis, this review will discuss the recent advances in allylic selective oxidation by different enzymes to reveal the great achievements and potentials in this field.

## Whole cell as a catalyst

### *Bacillus megaterium* CGMCC 1.1741

*Bacillus megaterium* is a rod-shaped, gram-positive prokaryotic bacterium, which has offered a green method to modify natural products (König et al., 2019). Zhang and his coworkers found that *Bacillus megaterium* CGMCC 1.1741 could transform the ursolic acid (2) into three oxidation products. For ursolic acid (2), introducing a hydroxyl group is a key step because it provides a functional group for subsequent modifications. In this study (Zhang et al., 2017), a very unique pathway for the biosynthesis of pentacyclic triterpenes was presented. However, the bioconversion of ursolic acid (2) gave lots of byproducts, and the yield of the desired product is not satisfactory (Figure 1D).

Zhang found that *Bacillus megaterium* CGMCC 1.1741 had the ability to catalyze pentacyclic triterpenes and steroids, which was the first report about the one biotransformation culture that could catalyze allylic oxidation on both Δ^12^-triterpenes and Δ^5^-steroids (Wang et al., 2020). In further studies, the aforementioned products were shown to be inhibitors of sterol biosynthesis (Schroepfer Jr, 1981; Berthier et al., 2004; Okabe et al., 2014; Gargiulo et al., 2017), which offered promising candidates for the prevention and treatment of cancers (Zhao et al., 2015). These findings not only broaden the substrate scope but also provide direct access to prepare these vital products with high stereoselectivity in one step (Figure 1E). Since P450 enzymes may be responsible for allylic hydroxylation, studies of gene clone and protein characterization of the potential P450s from *Bacillus megaterium* CGMCC 1.1741 is in process.

### Pleurotus sapidus

Berger’s group found that the white-rot fungus *Pleurotus sapidus* (PSA) could transform limonene into *cis/trans*-carveol and carvone (Onken and Berger, 1999). With further study on PSA, Holger Zorn and Wolfgang Maison proposed an effective way to transform valencene (9) into nootkatone (9a), which had a pleasant grapefruit-like aroma. Due to nootkatone (9a) being commonly used in the food and pharmaceutical industries, it is important that this type of compound is prepared in a biocatalytic manner (Fraatz et al., 2009; Krugener et al., 2010).

For example, the biocatalytic oxidation with PSA has a relatively wide range of substrates including several functionalized terpenoids such as *α*-pinene (10) and theaspirane (11) (Fraatz et al., 2009; Krugener et al., 2010; Rickert et al., 2012; Weidmann et al., 2015). In addition, PSA can also oxidize cyclohexene derivatives, but many reactions have limited regioselectivity, except that *tert*-butyl cyclohexene (14) was converted with good regioselectivity (Figure 1F).

Another point worthy of attention is that biocatalytic oxidations with PSA may be performed with the lyophilisate of the fungus. This provides a lot of convenience and is straightforward for the catalytic reaction. However, because the lyophilisate was used as a mixture of enzymes, oxidation of the substrate by pathways other than allylic hydroxylation might exist as the corresponding by-product was detected.

## Unspecific peroxygenases

Eighteen years ago, Pamela Manzi and coworkers found an enzyme that could oxidize halides and aryl alcohols, but the enzyme was not named (Ullrich et al., 2004). A few years later, additional aromatic, heterocyclic, and aliphatic substrates were found to be oxidized by this enzyme (Hofrichter and Ullrich, 2006; Ullrich and Hofrichter, 2007; Hofrichter and Ullrich, 2011), therefore, the name of this enzyme is called unspecific peroxygenase (UPO, EC1.11.2.1), which is also known as aromatic peroxygenase (APO). APO was discovered in the basidiomycetous fungus, *Agrocybe aegerita* (*Aae*UPO) (Ullrich and Hofrichter, 2005). UPOs were approached as a functional monoperoxygenase which can catalyze many reactions that involve the transfer of an oxygen atom by reacting with hydrogen peroxide. The reactions catalyzed by this enzyme range from epoxidation, hydroxylation, dealkylation, aromatization, sulfoxidation to dechlorination, and halide oxidation (Hofrichter and Ullrich., 2014; Hofrichter et al., 2015; Wang et al., 2017). For hydroxylation, in 2013, Peter and coworkers employed the whole cells to convert a series of alkenes into the corresponding epoxides and hydroxyl products in one step (Figure 1G). In this work (Peter et al., 2013), UPOs could insert oxygen into aliphatic and aromatic compounds that contain non-activated carbon atoms (stereoselectivity data is not given), although the reaction tends to form the epoxidation products except for chain alkenes, they found a broad spectrum of substrates catalyzed by the *Aae*UPO is another gift.

Moreover, many results showed that the hydroxylation reaction preferentially occurred in the free methylene groups (Gutiérrez et al., 2011; Peter et al., 2011; Kluge et al., 2012). On the other hand, UPOs have similar advantages to cytochrome P450 monooxygenases (CYP or P450) that can mediate allylic oxidation reactions. First of all, UPOs are usually extracellular and soluble proteins, while the P450s are usually intermembrane proteins that are relatively less stable. In addition, during the catalytic process, UPOs need H_2_O_2_ as a cosubstrate for the reaction, while the P450s usually require auxiliary electron transport systems and cofactors (Figure 1H).

**FIGURE 2:**
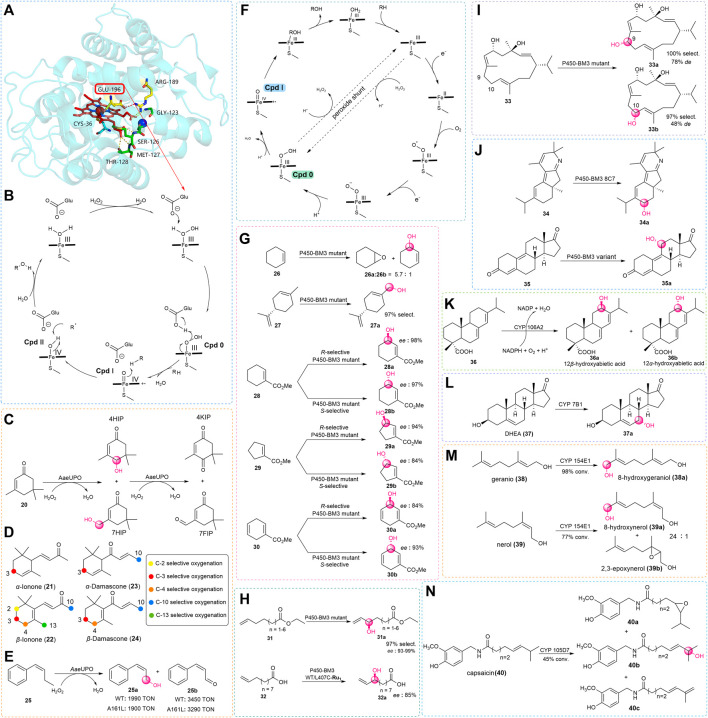
**(A)** Overall crystal structure of *Aae*APO (Wang et al., 2017). **(B)** Catalytic mechanism of *Aae*APO. **(C)** Isophorone hydroxylation and hyperoxidation catalyzed by *Aae*UPO. **(D)** Different colors indicate the positions where the substrate may be hydroxylated during UPO catalysis, obtaining the different oxygenated derivatives. **(E)** Bioconversion of the *Mth*UPO mutant A161L gains the allylic oxidation products. **(F)** Oxidation reaction mechanism of P450s. **(G)** Biocatalytic allylic oxidation of cyclic olefins by P450-BM3 mutants. **(H)** Biocatalytic allylic oxidation of chain terminal alkenes by P450-BM3 mutants. **(I)** Potential oxidation sites of *β*-cembrenediol (32) by the P450-BM3 mutant. **(J)** Hydroxylation reaction of terpenoids (33) and steroids (34) by P450-BM3 mutants. **(K)** Biocatalytic allylic oxidation by CYP106A2. **(L)** Biocatalytic allylic oxidation of DHEA (36) by CYP 7B1. **(M)** Biocatalytic allylic oxidation of gernio (37) and nerol (38) by CYP 154E1. **(N)** Biocatalytic allylic oxidation of capsaicin (39) by CYP 105D7.

Although the catalytic mechanisms of P450s and UPOs are similar, there are some fundamental structural differences at the enzyme active sites which are important to form compound I (Cpd I). After the crystal structure of *Aae*APO (Piontek et al., 2010) had been reported (Figure 2A), Hofrichter and coworkers studied the catalytic mechanism of the enzyme (Hofrichter and Ullrich., 2014). The structure has a heme-thiolate protein with acid-base residues of the distal glutamate 196 and arginine 189 at the active site. During the catalytic process, the iron-heme center activates the hydrogen peroxide first, then H_2_O_2_ reacts with the resting state UPO to form the compound 0 (Cpd 0). The complex is deprotonated by glutamate 196 by electron re-arrangement to give the key active Cpd I. Finally, this reactive species abstracts an H atom from the substrate to form a low degree of stereoselectivity for allylic hydroxylation reaction (Hrycay and Bandiera., 2012). In addition, UPOs are eukaryotic enzymes, and their expressions have been limited to yeasts in most circumstances (Wang, 2013; Molina-Espeja et al., 2014). In light of this, techniques of molecular biology have been used to resolve these issues, in particular the directed evolution.

Directed evolution is constituted of random or semi-rational mutagenesis which is extremely successful for improved enzyme abilities and has led to many enzyme libraries for different substrates (Bornscheuer et al., 2012; Hammer et al., 2017; Qu et al., 2020; Li B. B et al., 2021; Li D et al., 2021).

For one thing, due to the UPOs being eukaryotic enzymes and usually being glycosylated, most examples of heterologous expression appear to require expression in yeasts such as *Saccharomyces cerevisiae* and *Pichia pastoris* which are closely related to the microorganism that the enzyme was isolated from, for full activity. However, for wild-type UPOs, the yield of functional expression in yeast is low. Alcalde group used five rounds of directed evolution of the *Aae*UPO to resolve this issue, which resulted in the functional expression in *Saccharomyces cerevisiae* (Molina-Espeja et al., 2019). Since *Escherichia coli* (*E. coli*) has the characteristics of fast amplification, easy cultivation and low cost of cultivation, expression in the *E. coli* has become a good choice for most heterologous expressions in the laboratory (Baeshen et al., 2015). Wang (2016) successfully purified the wild-type *Aae*UPO by its expression in *E. coli*, however, the recombinant protein showed no activity, because of the formation of inclusion bodies (Wang, 2016). With the development of the directed evolution, Martínez and co-workers obtained *Collariella virescens* and *Daldinia caldariorum* from the ascomycetes by exploring the heterologous expression system of UPOs. They found the mutants of them to be active when expressed in *E. coli*, but the enzyme activity was low, with 38.18 U/mg and 7.68 U/mg, respectively (Linde et al., 2020).

Moreover, earlier results showed that wild-type UPOs catalyzed linear alkenes with a preference for allylic hydroxylation reaction but showed low chemoselectivity toward branched and cyclic alkenes for the formation of epoxide products. With the increasing number of newly discovered enzymes and screened substrates, some novel and interesting results were obtained. Gutiérrez and co-workers discovered that the UPOs were capable of selectively hydroxylating isophorone (20) to 4-hydroxyisophorone (4HIP) and 4-ketoisophorone (4KIP), which are now used in the flavor-and-fragrance and pharmaceutical industries (Aranda et al., 2019). They used three UPO enzymes, biocatalytic isophorone (20) with different selectivities. The first enzyme is *Aae*UPO, which is the earliest wild-type UPO described in previous reports. The enzyme isolated from *Marasmius rotula* aromatic peroxygenase (*Mro*APO) is another wild-type enzyme (Grobe et al., 2011). Last, the UPO of *Chaetomium globosum* (*Cgl*UPO) is a third wild-type peroxygenase (Kiebist et al., 2017). In this work, they found the *Aae*UPO had poor regioselectivity, and in addition to 4HIP, the reaction also formed 7-hydroxyisophorone (and 7-formylisophorone), yet this enzyme has the most stereoselectivity which provided the *S*-4HIP with an enantiomeric excess (*ee*) of 88% (Figure 2C). Then, this group tried some new substrates and found UPOs could accomplish the oxyfunctionalization of ionones and damascones. The products of these reactions are essential compounds for the flavor and important building blocks for pharmaceutical industries. Similar to the previous method, they used a series of UPOs to react with *α*-ionone (21), *β*-ionone (22), *α*-damascone (23), and *β*-damascone (24). In test, most types of UPOs showed high conversion, and oxidation of various positions was observed (Figure 2D). With *Aae*UPO, the products with the highest oxidation yield occurred at the C-3 position for *α*-Ionone (21), *α*-Damascone (23), and *β*-Damascone (24), and at the C-4 position for *β*-Ionone (22). With *Mro*UPO, the products with the highest oxidation yield occurred at the C-3 position for *α*-Ionone (21) and *α*-Damascone (23), and at the C-4 position for *β*-Ionone (22) and *β*-Damascone (24). With r*Cci*UPO, the products with the highest oxidation yield occurred at the C-3 position for *α*-Ionone (21), at the C-4 position for *β*-Ionone (22), and at the C-10 position for *α*-Damascone (23). For this enzyme with (24), oxidation seemed to occur with no preference, with a yield greater than 99% obtained for positions C-3, C-4, and C-10, respectively. With *Cgl*UPO, the products with the highest oxidation yield occurred at the C-3 position for *α*-Ionone (21) and *α*-Damascone (23), at the C-4 position for *β*-Ionone (22) and *β*-Damascone (24). With r*Dca*UPO, the products with the highest oxidation yield occurred at the C-3 position for *α*-Ionone (21) and *α*-Damascone (23), at the C-4 position for *β*-Ionone (22) and *β*-Damascone (24).

Although the only variation between the two types of ionone is the position of the double bond, the *α*-Ionone (21) demonstrated better regioselectivity (up to 99%) than the *β*-Ionone (22), possibly because of the preference of the UPOs toward the allylic substrate. This preference exists on *α*- and *β*-Damascones as well, however, the same enzyme’s regioselectivity was not consistent with ionones. Among the tested reaction, the hydroxylation of *Aae*UPO happened primarily at the terminal position of the butenoyl side chain, whereas *Cgl*UPO did not yield this product at all (Babot et al., 2020). The results of this study demonstrated the unique regioselectivity for diverse UPOs. Moreover, a little change in the structure of the substrate might lead to different regioselective products. Except for wild-type enzymes, in recent years a growing body of research has revealed the charm of directed evolution with high throughput screening. Weissenborn and co-workers used the variants of *Mth*UPO from *Myceliophthora thermophile* for enhancing the low selectivity with epoxidation of the double bond substrates such as cyclohexene and styrene. However, when methylstyrene (25) was used as the substrate (Knorrscheidt et al., 2021), it was surprising to find that the wild-type *Mth*UPO and one of its variants, A161L, had excellent selectivity for allylic oxidation. This unexpected result provided an idea for discovering enzymes with new catalytic performances for allylic oxidation in the future (Figure 2E).

## P450s

In recent decades, in order to comply with the principles of green chemistry, P450s are one of the most extensively studied biocatalysts. P450s are widely distributed in animals, plants, and microorganisms, which is a recognized multi-functional biological oxidation catalyst, and it has a wide source. The number of P450s from the bacteria has exceeded 62,000, and the number of P450s derived from the fungi has exceeded 85,000 species (Nelson, 2018). Furthermore, the enzymes catalyze the incorporation of molecular oxygen in the air into the substrate, which meets a green development. In addition, metalloenzymes have a heme-iron prosthetic group at the active site, which can overcome the barriers associated with the electronic structure of molecular oxygen (Jensen and Ryde., 2004). Although P450s have been shown to have great potential in biocatalysis, they require additional cofactors. In addition, they usually have the issues of poor stability and low solubility. These limit the industrial applications of enzymes. On one hand, with the development of catalytic self-sufficient P450, heme and reductase domains are fused in a polypeptide, which requires no additional redox partners that can avoid complex reaction conditions. On the other hand, substrates used for this field of study are generally hydrophobic, which usually causes substrate solubility issues, leading to relatively low transformation efficiencies. For this issue, several permeation methods are currently available, such as freeze and thaw, electropermeabilization, and using EDTA as a permeabilizer (Chen, 2007).

The activation of C–H bonds in allylic compounds has raised great interest in the chemical industry (Balcells et al., 2010). Due to the bonds in hydrocarbons being thermodynamically strong and usually kinetically inert, controlling the chemo-, regio-, and stereo-selectivities under mild conditions is rather difficult (Xue et al., 2017).

Considering these issues, one of the currently adopted methods for allylic oxidation is to use green and efficient enzyme catalysts, such as P450s (Urlacher and Girhard, 2019). It has been mentioned that the hydroxylation mechanism of UPOs and P450s is similar but is essentially different in terms of the formation of Cpd I (Figure 2F). In P450s, after the substrate binding to the enzyme active site, the heme iron accepts one electron of the cofactor to be reduced to Fe^2+^. Then the reduced heme iron reacts with molecular oxygen to generate a complex, which is reduced by another single electron and subsequently protonated to give Cpd 0. Then, the cleavage of the O–O bond brings in a water molecule to give the highly reactive Cpd I. Finally, Cpd I oxidizes the substrate which leads to the formation of the hydroxylation product and restores the resting state of the heme. In addition, some P450s can utilize the H_2_O_2_ as an oxygen source and promote the formation of Cpd I *via* the peroxide shunt pathway (Munro et al., 2013). Similar to the UPOs, this enzyme is also relatively sensitive to H_2_O_2_, which should be considered in reaction.

Miura and Fulco (1974) identified CYP102A1 from *Bacillus megaterium*, wildly known as fatty acid hydroxylase Cytochrome P450-BM3, as the first enzyme of this family in 1970 and has been an extensively studied self-sufficient P450 in the past few decades (Miura and Fulco., 1974). More and more studies on allylic hydroxylation have been described based on the crystal structure of P450-BM3 (Whitehouse et al., 2012). In the early stage, when Arnold’s group looked into alkene epoxidation (Farinas et al., 2004), they discovered that the mutant of the P450-BM3 not only obtained the epoxide product but could also catalyze allylic hydroxylation of cyclohexene (Figure 2G). As early as 1993, Montellano had discovered that when the wild-type P450-BM3 was used, oxidation of *ω*-unsaturated fatty acids preferentially occurred at its allylic position.

Therefore, in addition to cyclic alkenes, Arnold also tried to react linear alkenes with this mutant and found that only allyl hydroxylated products were produced (Shirane et al., 1993). In view of this, there are more instances of P450-BM3 and variants catalyzed oxidation of the allylic site of natural product to acquire beneficial hydroxylated building blocks of pharmaceuticals and intermediates. Limonene (27) being the cheap and promptly accessible terpene, Pleiss and co-workers used variants of P450-BM3 to convert (4*R*)-limonene to perillyl alcohol (27a) which might slow down the process of tumor development in the pancreatic, breast, colon, and liver (Seifert et al., 2011). The anti-tumor substance can be obtained by the selective C-7 hydroxylation of limonene (27). However, owing to the substrate that could react with many active positions, perillyl alcohol (27a) was not detected when using the wild-type P450-BM3 (Seifert et al., 2009). After iterative rounds of molecular modeling (Seifert et al., 2011), mutagenesis, and screening, a triple mutant (A264V/A328V/L437F) with high regioselectivity of 97% for the purpose of conversion of limonene (27) to perillyl alcohol (27a) was identified (Figure 2G). A few years later, Reetz and his colleague started mutating from the site near the active center of P450-BM3 and combined with the method of iterative saturation mutation to provide a high degree of regio- and enantio-selectivity. In this work, they reported the first example of P450-BM3 and variants acting as catalysts for the selective complementary synthesis hydroxylation of templates and other similar substrates. For cyclohexene-L-carboxylic acid methyl ester (28), they accomplished the goal of obtaining the mutant (F87L/A328V) with *R* selectivity of 98% and the mutant (A328S) with *S* selectivity of 97%, moreover, both mutants can achieve >95% substrate conversion under specific reaction condition. Next, they obtained mutants by screening from the established combinatorial library (Agudo et al., 2012), some of these P450 mutants could also be used to stereoselectively hydrolyze a structurally similar cyclopentene (29) and cyclohexadiene (30) molecule with 84%–94% *ee* (Figure 2G). Except for cyclic olefins, the Pietruszka group identified the mutant (A74G/L188Q) of P450-BM3 as a highly selective biocatalyst for the C–H oxidation of linear terminal olefins (31) with regio- (90%) and stereo-selectivity (93%–99% *ee*) by utilizing a comparative method based on the directed evolution (Neufeld et al., 2014). Around the same time, with the introduction of some new technologies, Cheruzel and co-workers reported an efficient combination of light-driven and P450-BM3 enzymes, this method could cause selective oxidation of 10-undecenoic acid (32) with *R* enantiomer in 85% *ee*. In addition (Figure 2H), there were no products formed if there was no light source or reductive quencher involved (Kato et al., 2014).

With the continuing exploration of P450-BM3, Urlacher and co-workers demonstrated the sufficient selective oxidation of the tobacco cembranoid, *β*-cembrenediol which can inhibit tumor promotion by the use of tetradecanoylphorbol acetate by inhibiting the early antigen of the Epstein–Barr virus. The compound was a 14-membered macrocycle (33), which contains seven potential sites for allylic hydroxylation along with three epoxidation sites. However, the Urlacher group still obtained a few P450-BM3 mutants (Figure 2I) that allowed for the selective hydroxylation of the adjoining position C-9 with 100% regioselectivity and 78% diastereoselectivity for the mutant (F87A/I263L) and C-10 with 97% regioselectivity and 48% diastereoselectivity for the mutant (L75A/V78A/F87G) (Le-Huu et al., 2015).

In recent years, examples of biocatalysis for the total synthesis of drugs have gradually increased. Stoltz and coworkers made use of the engineered P450-BM3 enzyme for the total synthesis of nigelladine A, which is the natural product that belongs to the diterpenoid alkaloids. The construction of the ketone carbonyl (34) at the C-7 position is a challenging step because the hydroxylation of the 2^o^ carbon at the C-7 position is accompanied by the competitive process of the 3^o^ carbon at the C-10 position. The optimal mutant 8C7 was obtained by directed evolution of P450-BM3, with residues 75 and 181 being to alanine (Figure 2J). Finally, an ideal product in a 2.8:1 ratio at the desired position was obtained (Loskot et al., 2017). Aitao Li and coworkers designed a two-step chemoenzymatic strategy to generate the steroid drug Trenbolone (35a) (Peng et al., 2022). In this study, the P450-BM3 mutant LG23/T438S was used as a key enzyme to catalyze estra-4,9-diene-3,17-dione (35) C-11 hydroxylation with 94% selectivity and >95% conversion. It should be noted that this was the only bacterial C-11 selective P450 for the model substrate (Figure 2J), others were mainly reported using filamentous fungi with unsatisfactory selectivity and activity (Carballeira et al., 2009).

Those cases show the strong specificity of the P450-BM3. Other types of P450s have also contributed to allylic hydroxylation of various substrates in recent decades. In 2011, Bernhardt reported the prokaryotic cytochrome P450 CYP106A2 from *Bacillus megaterium* ATCC 13368 which was discovered to be the first bacterial P450 diterpene hydroxylase capable of regioselectively allylic hydroxylation of abietic acid (36) (Figure 2K) in a single step (Bleif et al., 2011). Afterward, to address the insufficient supply of cofactors, P450 CYP7B1 was used by Song in a bioelectrocatalytic 7*α*-hydroxylation of dehydroepiandosterone (DHEA, 37). To regenerate cofactors, Zhang et al. (2019) used an electricity-driven NADPH regeneration with a concomitant electron shuttle. By using this system, 289 ± 8 mg·L^−1^ of 7*α*-hydroxy-DHEA (37a) was produced (Figure 2L). In addition to the steroid compound, Urlacher and coworkers exploited the use of acyclic terpenoids as a substrate and found there was a sort of CYP154E1 from *Thermobifida fusca* YX that enables this kind of hydroxylation. They discovered that (Bogazkaya et al., 2014) the wild-type CYP154E1 could catalyze the selective oxidation of natural products geraniol (38) and nerol (39). When the wild-type strain reacted with geraniol (38), it was more prone to allylic hydroxylation to generate a single product of 8-hydroxygeraniol, and the reaction conversion was as high as 98%. However, when the wild-type CYP154E1 was used for nerol (39), the yield of conversion was slightly lower. In addition to 8-hydroxygeraniol (39a), 2,3-epoxynerol (39b) was also produced (Figure 2M). The importance of the hydroxylated norisoprenoids has been mentioned in the previous description of the oxyfunctionalization of UPOs. Bernhardt used these types of compounds as substrates for the exploration of the oxidative activity of myxobacterial CYP260B1 and CYP267B1 from *Sorangium cellulosum* Soce56. The study found the regioselective hydroxylation at the allylic position by CYP260B1 toward a single product is lower compared to that by CYP267B1, as CYP267B1 showed only hydroxylation activity for all tested substrates, while CYP260B1 could also catalyze epoxidation reactions to form byproducts (Litzenburger and Bernhardt., 2016).

Based on the metabolism studies of capsaicin (40) by Tian et al. (2019) found the P450 enzyme CYP105D7 from *Streptomyces avermitilis* could be a catalyst for the oxidation of the capsaicin (40), which is a pain reliever and also has activities of anti-tumor, anti-obesity, and cardio-protection. In this reaction (Figure 2N), three products were generated, with the majority being obtained by *ω*-1 allylic hydroxylation at a yield of 64% (40b), the alkyl dehydrogenation product at a yield of 23% (40c), and the epoxidation product at a yield of 13% (40a) (Ma et al., 2021).

## Conclusion and outlook

As the demand for oxygenated allylic groups in food, fragrance, cosmetics, pharmaceutical, and materials has grown, so has their significance. Several strategies for the oxidation of allyl to allyl alcohols or allyl esters have been published in recent years. However, compounds often have multiple allylic positions, and the requirement of specificity and chirality is challenging to traditional organic chemistry. Since biocatalysis is emerging to be a viable alternative to traditional chemical catalysis, the use of biocatalytic oxyfunctionalization reactions has increased over the past decade, including whole-cell as the catalyst, UPO enzyme, and P450 enzyme presented in this review. Among the majority of examples presented the whole-cell as the catalyst was often difficult to have high selectivity and yield. The UPO-mediated catalysis was usually accompanied by hydroxylation and epoxidation products, but for this problem, the mutants of UPO have improved chemical selectivity. Being sensitive to hydrogen peroxide and difficult to express in *E. coli* has limited its wide application in industries. Compared with them, the P450 enzyme is relatively easy to express in *E. coli* and the mutants generally have better selectivity and activity. Particularly the engineered self-sufficient P450s that have been discussed in this review could catalyze a series of useful reactions, could present promising potential, and broad application prospects. However, the poor stability and the limited scope of substrate usually are the main hinders to their industrial application. With the discovery of new enzymes and the rapid development of directed evolution, substrate engineering, synthetic biology, and immobilization, researchers can continue to address these bottlenecks in the future. In summary, given the advances in enzyme development, biocatalysts can still be considered to have considerable potential for chemo-, regio- and stereo-selectivity oxygenation reactions.
